# Anti-inflammatory/anti-amyloidogenic effects of plasmalogens in lipopolysaccharide-induced neuroinflammation in adult mice

**DOI:** 10.1186/1742-2094-9-197

**Published:** 2012-08-13

**Authors:** Masataka Ifuku, Toshihiko Katafuchi, Shiro Mawatari, Mami Noda, Kiyotaka Miake, Masaaki Sugiyama, Takehiko Fujino

**Affiliations:** 1 Department of Integrative Physiology, Graduate School of Medical Sciences, Kyushu University, Fukuoka 812-8582, Japan; 2 Institute of Rheological Function of food, Kasuya-gun, Fukuoka 811-2501, Japan; 3 Laboratory of Pathophysiology, Graduate School of Pharmaceutical Sciences, Kyushu University, Fukuoka 812-8582, Japan; 4 Central Research Institute, Marudai Food Co. Ltd, Osaka 569-8577, Japan

**Keywords:** Neuroinflammation, Phospholipids, Microglia, Alzheimer’s disease

## Abstract

**Background:**

Neuroinflammation involves the activation of glial cells in neurodegenerative diseases such as Alzheimer’s disease (AD). Plasmalogens (Pls) are glycerophospholipids constituting cellular membranes and play significant roles in membrane fluidity and cellular processes such as vesicular fusion and signal transduction.

**Methods:**

In this study the preventive effects of Pls on systemic lipopolysaccharide (LPS)-induced neuroinflammation were investigated using immunohistochemistry, real-time PCR methods and analysis of brain glycerophospholipid levels in adult mice.

**Results:**

Intraperitoneal (i.p.) injections of LPS (250 μg/kg) for seven days resulted in increases in the number of Iba-1-positive microglia and glial fibrillary acidic protein (GFAP)-positive astrocytes in the prefrontal cortex (PFC) and hippocampus accompanied by the enhanced expression of IL-1β and TNF-α mRNAs. In addition, β-amyloid (Aβ_3–16_)-positive neurons appeared in the PFC and hippocampus of LPS-injected animals. The co-administration of Pls (i.p., 20 mg/kg) after daily LPS injections significantly attenuated both the activation of glial cells and the accumulation of Aβ proteins. Finally, the amount of Pls in the PFC and hippocampus decreased following the LPS injections and this reduction was suppressed by co-treatment with Pls.

**Conclusions:**

These findings suggest that Pls have anti-neuroinflammatory and anti-amyloidogenic effects, thereby indicating the preventive or therapeutic application of Pls against AD.

## Background

It has been demonstrated in mice and rats that the systemic administration of lipopolysaccharide (LPS) and polyriboinosinic:polyribocytidylic acid (poly I:C), ligands for toll-like receptor (TLR) 4 and TLR 3, respectively, induce neuroinflammation in the central nervous system (CNS), thus leading to neurodegeneration, the suppression of neurogenesis and the impairment of cognitive behavior [1-3]. One of the possible mechanisms of neuroinflammation may be the production of β-amyloid proteins (Aβ). For example, a single intraperitoneal (i.p.) injection of LPS increases the activity of β-secretase, a key rate-limiting enzyme that initiates Aβ formation, and the concentration of brain Aβ_1–42_ in adult but not young mice [1]. Furthermore, the intracellular accumulation of Aβ_1–42_ in hippocampal pyramidal neurons following daily injections of LPS for seven days has been immunohistochemically demonstrated [1]. Although the precise mechanisms underlying LPS-induced amyloidogenesis have not yet been determined, it is likely that proinflammatory cytokines such as IL-1β, TNF-α, IFN-γ, and reactive oxygen/nitrogen species (ROS/RNS) released from activated glial cells play significant roles in Aβ formation, which are suppressed by NSAIDs through the activation of peroxisome proliferator-activated receptor-γ (PPAR-γ) [4-6].

Plasmalogens (Pls) are unique glycerophospholipids that contain a vinyl ether bond at the *sn*-1 position of the glycerol moiety. They are found in all mammalian tissues, especially in the heart and brain, in which ethanolamine Pls (PlsEtn) are much more abundant than choline Pls (PlsCho) [7]. Pls release either docosahexaenoic acid (DHA) or arachidonic acid (ARA) from the *sn*-2 position through the activation of Pls-selective phospholipase A_2_ (Pls-PLA_2_) [8,9]. Pls are not only structural membrane components and reservoirs for second messengers, but they are also involved in membrane fusion, ion transport and cholesterol efflux [7]. In addition, the vinyl ether bond at the *sn*-1 position makes Pls more susceptible to oxidative stress than corresponding ester bonded glycerophospholipids, which act as antioxidants and protect cells from oxidative stress [10-13].

It has been shown that patients suffering from Alzheimer’s disease (AD) have reduced PlsEtn levels in the cortex and hippocampus [14-16]. The reduction of PlsEtn levels seems to be specific since other neurodegenerative diseases such as Huntington’s and Parkinson’s do not show decreases in corresponding affected brain regions (the caudate nucleus and the substantia nigra, respectively) [7,14,17]. Furthermore, circulating PlsEtn levels are also decreased depending on the severity of dementia [18,19]. It has been suggested that deficiencies of PlsEtn may lead to increases in the vulnerability of neural membranes to oxidative stress, destabilization of membranes, impairment of muscarinic cholinergic signals and abnormal amyloid precursor processing [7,17,20].

Although Pls are considered to be involved in the pathology of AD, the influence of Pls on Aβ accumulation has not been examined, probably due to the difficulty of extracting massive amounts of intact Pls. Recently, we developed a new method for preparing highly pure Pls [21], which enabled us to investigate this issue. In the present study, in order to elucidate the anti-neuroinflammatory/anti-amyloidogenic actions of Pls, we investigated the effects of co-administered Pls on the systemic LPS-induced changes in the morphology of glial cells, the expression of cytokines, the accumulation of Aβ and the levels of Pls in the prefrontal cortex (PFC) and hippocampus of adult mice.

## Methods

All experimental procedures involving the use of animals were approved by the Ethics Committee on Animal Experiments at Kyushu University and were in accordance with the Guiding Principles for the Care and Use of Animals of the Physiological Society of Japan. All efforts were made to minimize animal suffering and the number of animals used in the study.

### Animals

Male C57/6J mice weighing 32 to 37 g (10 months old) were used in all experiments. The animals were housed five per cage at a temperature of 22 ± 2°C with 12 hour light/12 hour dark cycles (lights on at 8:00) with free access to laboratory food and water. The mice were randomly divided into four groups: control (Con), LPS, LPS + Pls and Pls. LPS (Sigma-Aldrich, St. Louis, MO, USA) was dissolved in saline, while the Pls were dissolved in corn oil then sonicated to ensure complete solubilization. The LPS group received an i.p. injection of LPS (250 μg/kg) followed by corn oil in the morning (9:00 to 10:00) daily for seven days (days 1 to 7). The LPS + Pls group was treated with LPS followed by Pls (20 mg/kg) for seven days, while the Pls group was injected with saline and Pls. The Con group was given saline and corn oil for seven days. All animals were sacrificed on day 8. The body weights were measured in the morning before injection on day 1 to day 8.

### Pl preparation

The Pls used in the present study were prepared from chicken breast muscle using a previously reported method [21]. A HPLC used for phospholipid separation [22] indicated that the purified Pls consisted of 47.6% PlsEtn, 49.3% PlsCho, 2.4% sphingomyelin (SM) and 0.5% other phospholipids. The composition of fatty acids of PlsEtn and PlsCho was analyzed using the previously described HPLC method [21], as shown in Table 1.

**Table 1:** The fatty acid composition of the PlsEtn and PlsCho in the purified Pls

**Numerical symbol**		**PlsEtn (%)**	**PlsCho (%)**
16:0	(palmitic acid)	3.6	20.5
18:0	(stearic acid)	2.2	12.4
18:1, *n*-9	(oleic acid)	26.3	20.1
18:2, *n*-6	(linoleic acid)	4.1	10.1
18:3, *n*-3	(α-linolenic acid)	7.2	3.8
20:4, *n*-6	(arachidonic acid, ARA)	24.9	17.2
22:6, *n*-3	(docosahexaenoic acid, DHA)	18.6	2.3
others		13.2	13.6

### Immunohistochemistry and immunofluorescence

The mice were deeply anesthetized with pentobarbital (50 mg/kg) and transcardially perfused with PBS followed by 4% paraformaldehyde. For each animal, the brain was removed, post-fixed for 24 hours and then transferred successively to 20% and 30% sucrose solutions. Subsequently, the brains were frozen on a cold stage and sliced into 30 μm thicknesses using a cryostat. The sections were permeabilized with 0.3% Triton-X 100 (Sigma-Aldrich) in PBS for 15 minutes and blocked in PBS containing 1% BSA and 5% normal donkey serum (Jackson ImmunoResearch Lab., West Grove, PA, USA) for 60 minutes at room temperature. The sections were incubated in blocking solution (Block Ace, Dainippon Pharmaceutical, Osaka, Japan) for 30 minutes at room temperature and then incubated with rabbit polyclonal antibodies against Iba-1 (1:10000; Wako Pure Chem. Indus., Osaka, Japan), which are known to have specific affinity for microglial Ca^2+^-binding proteins and are highly expressed by activated microglia, and anti-glial fibrillary acidic proteins (GFAPs, 1:3000; Sigma-Aldrich) for astrocytes in 10% Block Ace in PBS at 4°C overnight. Other sections were incubated with polyclonal antibodies against Aβ_3–16_ (1:1000; ab14220, Abcam, Cambridge, UK) and NeuN (1:1000; Millipore, Billerica, MA, USA). According to the manufacturer’s instructions, ab14220s react with all isoforms of mouse and rat Aβ. The rinsed sections were incubated for six hours with Alexa Fluor 488 goat anti-rabbit IgG or Alexa Flour 568 goat anti-mouse immunoglobulin G (IgG) (1:1000; Invitrogen, Eugene, OR, USA) at room temperature. Every treatment was followed by washing three times for five minutes with PBS. The sections were then mounted in the perma fluor aqueous mounting medium (Thermo Fisher Scientific, Waltham. MA, USA).

### Quantitative analysis of fluorescence intensity

All samples were analyzed with a confocal laser-scanning microscope (LSM510 Meta; Carl Zeiss, Jena, Germany). The number of glial cells in 90 to 100 areas of 200 μm x 200 μm in four slices per brain was counted and the average number/4 x 10^4^ μm^2^ was obtained for each brain.

### Real-time PCR

Mice were deeply anaesthetized with pentobarbital and perfused transcardially with phosphate buffered saline, then the PFC and hippocampus were removed immediately. Total RNA was isolated from the samples using magnetic beads (MagExtractor system; Toyobo, Tokyo, Japan) after homogenizing the tissues. Primer pairs were chosen to flank at least one intron. The amount of total RNA was quantified by measuring the optical density 250 using a Nanodrop spectrophotometer (Nanodrop, Wilmington, DE, USA). For reverse transcription, 100 ng of total RNA was transferred to the reaction using an RNA PCR kit (AMV) (Takara Bio Inc., Ootsu, Japan) and 9-mer random primers. SYBR-Green real-time PCR (Applied Biosystems, Foster City, CA, USA) was performed on cDNA prepared from each sample using the THUNDERBIRD SYBR qPCR Mix, ROX reference dye (Toyobo) and 0.5 mM of each primer. The data were analyzed using 7500 System software v2.0 (Applied Biosystems). All values of cytokines were normalized to the corresponding β-actin concentration obtained using the same method. The sequences of primers were follows: IL-1β, sense; 5′- CTCCATGAGCTTTGTACAAGG -3′, antisense; 5′- TGCTGATGTACCAGTTGGGG -3′; TNF-α, sense; 5′- CCACCACGCTCTTCTGTCTAC -3′, antisense; 5′- TGGGCTACAGGCTTGTCACT -3′ β-actin, sense; 5′- TTGCTGACAGGATGCAGAAGGAG -3′, antisense; 5′- GTGGACAGTGAGGCCAGGAT -3′. The predicted sizes of the PCR products were 245 bp for IL-1β, 196 bp for TNF-α, and 127 bp for β-actin mRNA.

### Measurement of the Pl levels in the PFC and hippocampus

Mice were deeply anesthetized with pentobarbital (50 mg/kg) and transcardially perfused with sterile PBS. For each animal, the brain was removed and the PFC and hippocampus were dissected in a dish filled with ice-cold PBS. The samples (300 mg to 500 mg) were stored at −80°C until Pl measurement. Total lipids were extracted using the method of Folch and coworkers [23], and the relative composition of phospholipid classes, including Pls, was measured as previously reported [22].

### Statistical analysis

The results are expressed as the mean ± SEM. The body weights (BWs), numbers of Iba-1^+^ and GFAP^+^ cells and amounts of mRNAs were compared using one-way analysis of variance (ANOVA) followed by post hoc (Scheffe’s) test. Changes in the PlsEtn levels and the ratio of PlsEtn/Phosphatidyl Etn (PEtn) determined after LPS and Pl injection were evaluated using the non-parametric Kruskal-Wallis test followed by the Steel test for multiple comparisons. Values of *P* <0.05 were considered to be statistically significant.

## Results

### Body weight changes after LPS and Pls

**Figure 1:**
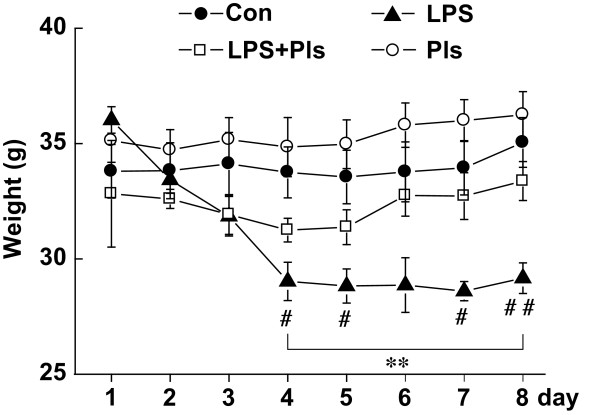
**Body weight (BW) changes after LPS/Pls administration.** BW was measured immediately prior to injection on days 1 to 7 and on day 8 before sacrifice. Filled circle: Con group; open circle: Pls group; filled triangle: LPS group; and open rectangle: LPS + Pls group. Each group, n = 8, **, *P* <0.1, LPS versus Pls group; #, *P* <0.05 and ##, *P* <0.01, LPS versus Con group. Con, control; LPS, lipopolysaccharide; Pls, plasmalogens.

The BWs of the mice in the LPS group started to decrease on day 2 and showed significant differences between groups on day 4 that lasted until day 8 (day 4: F(3,28) = 7.1, day 5: F(3,28) = 8.1, day 6: F(3,28) = 6.0, day 7: F(3,28) = 9.0 and day 8: F(3,28) = 9.4, *P* <0.01, respectively, each group, n = 8). The post hoc test indicated that the BWs of the LPS group were different from those of the control (Con) group (day 4, 5, 7 and 8, *P* <0.05) and the Pls group (from day 4 to 8, *P* <0.01). However, the LPS + Pls group showed no significant differences between either the Con or Pls group in terms of BW at any point (Figure 1).

### Suppression of glial activation by Pls

**Figure 2:**
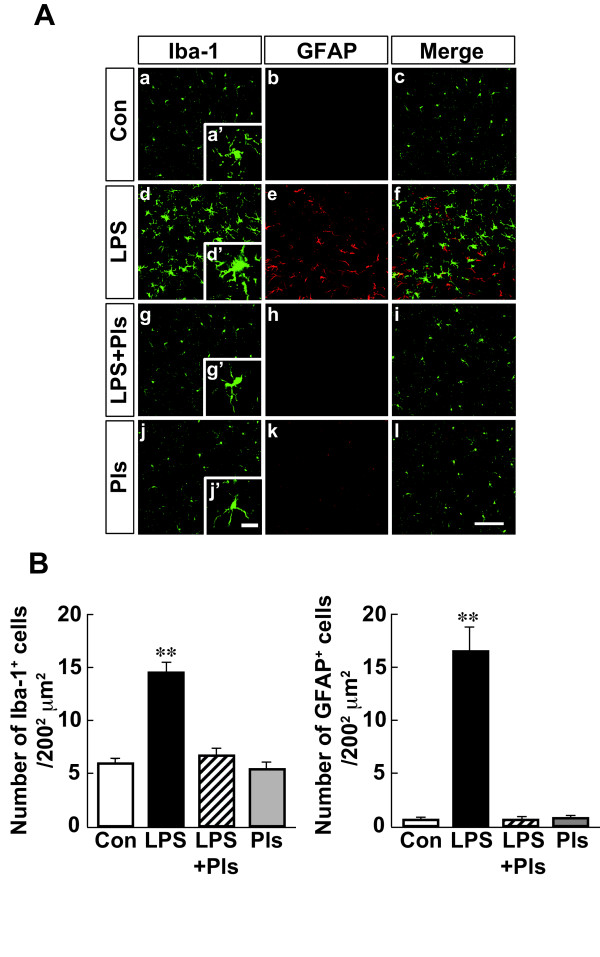
**Activation of glial cells in the murine PFC following LPS injection (i.p.) performed on seven consecutive days and suppression by Pls applied immediately after each LPS injection. A**, Iba-1-positive microglia (green) and GFAP-positive astrocytes (red). The number and intensity of immunoreactivity of microglia increased after LPS treatment (d) with hypertrophy (d’) compared with that observed in the Con group (a and a’) and was suppressed by application of Pls (g and g’). The Pls group (j and j’) showed no differences from the Con group. GFAP-positive astrocytes also demonstrated increases in number and intensity due to LPS and suppression by Pls (middle column). Iba-1 and GFAP immunostaining did not merge with each other (f). Scale bar: low magnification, 100 μm, and high magnification, 20 μm. **B**, A summary of LPS-induced increases in the numbers of microglia (left) and astrocytes (right) and suppression by Pls (each bar, n = 8). **, *P* <0.01, respectively. Con, control; GFAP, glial fibrillary acidic protein; LPS, lipopolysaccharide; PFC, prefrontal cortex; Pls, plasmalogens.

As shown in Figure 2Aa, the Con group that received saline and corn oil for seven days showed typical features of Iba-1-positive (green) resting microglia with small and compact soma bearing ramified processes (a’) in the PFC. GFAP was immunostained with weak fluorescence (red) in astrocytes (b). However, the i.p. administration of LPS (250 μg/kg/day) for seven days (LPS group, second row) resulted in neuroinflammation showing increased numbers of Iba-1-positive microglia and intense immunoreactivity (d) with activated phenotypes of marked cellular hypertrophy and retraction of cytoplasmic processes (d’). GFAP-positive astrocytes also increased in number and intensity (e). As shown in Figure 2Ag and h, the increases in the number of activated microglia and astrocytes in the PFC were suppressed by i.p. administration of LPS and Pls (20 mg/kg) (LPS + Pls group). Iba-1-positive microglia and GFAP-positive astrocytes did not merge with each other in all groups (c, f, i, and l). Figure 2B shows a summary of the LPS-induced increases in the number of glial cells and the suppression of this increase by Pls (each bar, n = 8). The number of microglia (left) and astrocytes (right) significantly increased following LPS injection (F(3,28) = 38.4, *P* <0.01; F(3,28) = 45.8, *P* <0.01, respectively). The multiple-range test indicated that the numbers of microglia and astrocytes in the LPS group were different from those in the Con, Pls, and LPS + Pls groups (Scheffe’s test, *P* <0.01, respectively), while the LPS + Pls group did not differ from the Con or Pls groups for either microglia or astrocytes.

**Figure 3:**
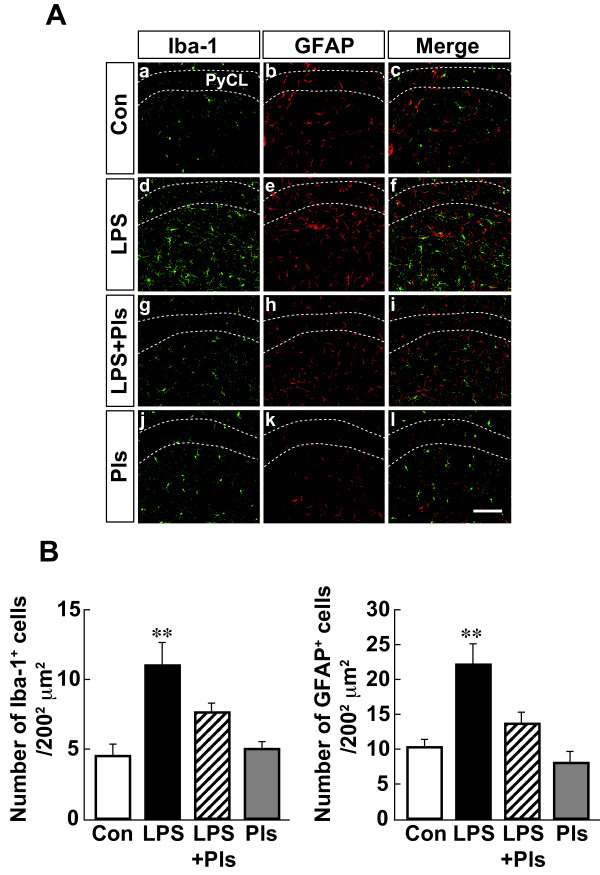
**Activation of glial cells following LPS (i.p.) injection and suppression by Pls in the murine CA1 region of the hippocampus. A**, Iba-1-positive microglia (green) and GFAP-positive astrocytes (red). The number and intensity of immunoreactivity of microglia and astrocytes increased following LPS treatment (d and e) compared with that observed in the Con group (a and b) and was suppressed by Pls (g and h). They did not merge with each other (right column). PyCL: pyramidal cell layer of the hippocampus. Scale bar: 100 μm. **B**, A summary of LPS-induced increases in the numbers of microglia (left) and astrocytes (right) and suppression by Pls (each bar, n = 8). **, *P* <0.01. Con, control; GFAP, glial fibrillary acidic protein; i.p., intraperitoneal; LPS, lipopolysaccharide; Pls, plasmalogens.

In the CA1 region of the hippocampus, both Iba-1-positive microglia and GFAP-positive astrocytes increased in number in the LPS group (Figure 3Ad and e) compared with that observed in the control group (a and b). Similar to that observed in the PFC, the increases in the number of activated glial cells were attenuated following the administration of Pls (g and h). As shown in Figure 3B, the statistical analysis indicated significant differences in the numbers of microglia (left: F(3,28) = 10.7, *P* <0.01) and astrocytes (right: F(3,28) = 11.2, *P* <0.01) between the groups (each bar, n = 8). LPS injection increased the numbers of microglia (left: *P* <0.01) and astrocytes (right: *P* <0.01). The increases were significantly suppressed by Pls to the levels of microglia and astrocytes observed in the Con and Pls groups (LPS + Pls group, *P* <0.05, respectively).

**Figure 4:**
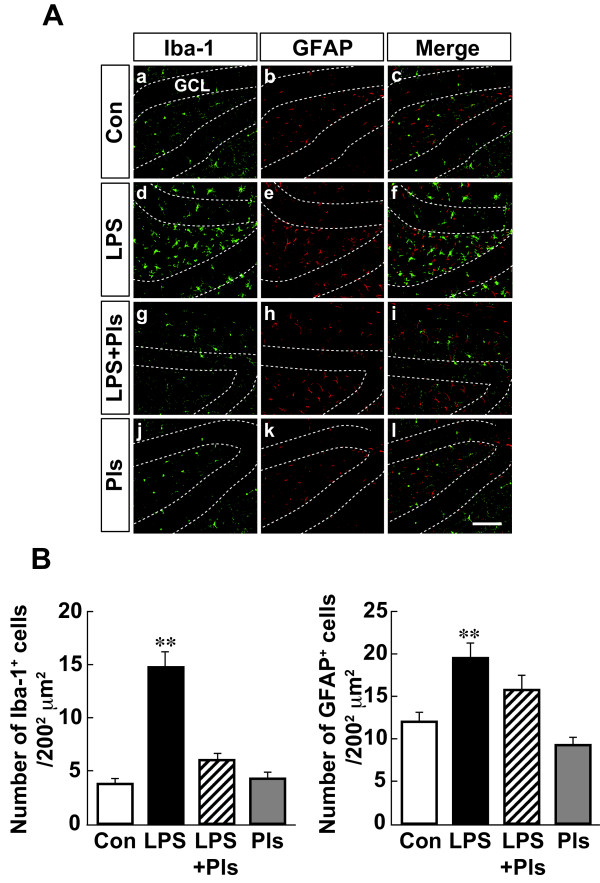
**Activation of glial cells following LPS (i.p.) injection and suppression by Pls in the murine dentate gyrus of the hippocampus. A**, Iba-1-positive microglia (green) and GFAP-positive astrocytes (red). The number and intensity of immunoreactivity of microglia and astrocytes increased following LPS treatment (d and e) compared with that observed in the Con group (a and b) and was suppressed by Pls (g and h). They did not merge with each other (right column). GCL: granule cell layer. Scale bar: 100 μm. **B**, A summary of LPS-induced increases in the numbers of microglia (left) and astrocytes (right) and suppression by Pls (each bar, n = 8). **, *P* <0.01. Con, control; GFAP, glial fibrillary acidic protein; i.p., intraperitoneal; LPS, lipopolysaccharide; Pls, plasmalogens.

As shown in Figure 4A, the dentate gyrus (DG) of the hippocampus also demonstrated LPS-induced increases in the number and intensity of immunostaining for Iba-1 in microglia (d) and GFAP in astrocytes (e) compared with that observed in the Con group (a and b). These increases were suppressed by simultaneous injection of Pls (g and f, respectively). One-way ANOVA indicated significant differences in the numbers of microglia (Figure 4B, left: F(3,28) = 41.4, *P* <0.01) and astrocytes (right: F(3,28) = 11.5, *P* <0.01) between the groups (each bar, n = 8). The differences in the numbers of microglia and astrocytes in the Con and Pls groups (*P* <0.01) and the LPS + Pls group were significant (*P* <0.01 and *P* <0.05, respectively).

### Suppression of LPS-induced increases in cytokine mRNA by Pls

**Figure 5:**
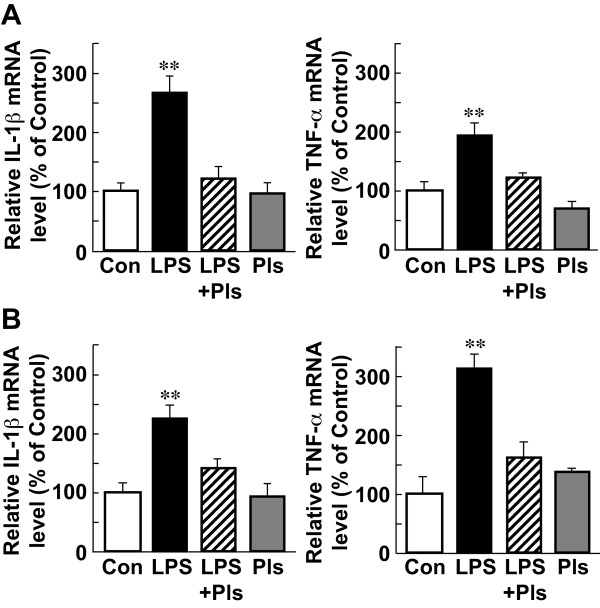
**Increases in the levels of IL-1β and TNF-α mRNAs following LPS (i.p.) injection and suppression by Pls in the murine PFC and hippocampus. A**, Significant increases in the levels of IL-1β (left) and TNF-α (right) mRNAs in the PFC were seen in the LPS group compared with that observed in the other groups (Con, LPS + Pls and Pls groups). **B**, Significant increases in the levels of IL-1β (left) and TNF-α (right) mRNAs in the hippocampus were seen in the LPS group compared to that observed in the other groups. Each bar, n = 5, **, *P* <0.01. _Con, control; IL-1β, interleukin-1β; i.p., intraperitoneal; LPS, lipopolysaccharide; PFC, prefrontal cortex; Pls, plasmalogens; TNF-α, tumor necrosis factor α.

As shown in Figure 5A, the relative amounts of mRNAs for IL-1β (left) and TNF-α (right) in the PFC were significantly different (ANOVA test, F(3,16) = 19.3, *P* <0.01, and F(3,16) = 14.9, *P* <0.01, respectively) between the groups (each bar, n = 5). The post hoc test indicated that the levels of both IL-1β and TNF-α mRNAs in the LPS group were significantly higher than those observed in the other groups (Scheffe’s test, *P* <0.01, respectively), while the levels in the LPS + Pls group were not different from those observed in the Con and Pls groups, suggesting that Pls have a suppressive effect on LPS-induced cytokine expression (Figure 5A).

Similar to the findings observed in the PFC, both cytokine mRNAs increased in the hippocampus in the LPS group (left: IL-1β, F(3,16) = 10.1, *P* <0.01; right: TNF-α, F(3,16) = 17.7, *P* <0.01) in comparison to that observed in the other three groups (Figure 5 B, *P* <0.01, respectively; each bar, n = 5).

### Suppression of LPS-induced Aβ accumulation by Pls

**Figure 6:**
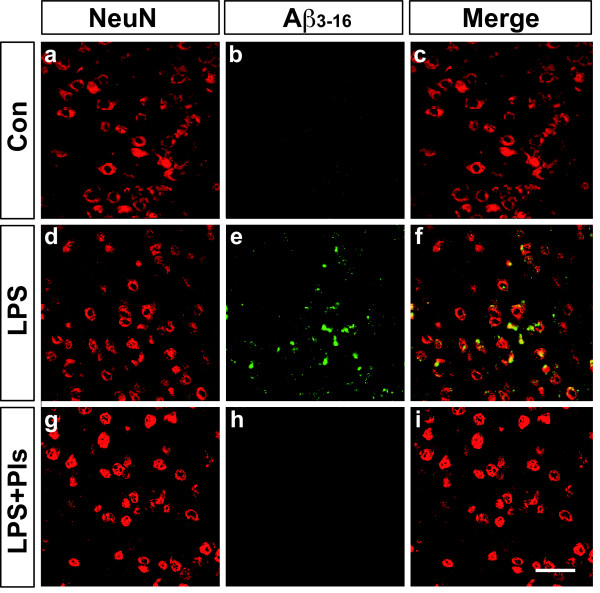
**Accumulation of Aβ proteins following LPS (i.p.) injection and suppression by Pls in the murine PFC.** Neurons were stained with NeuN (red). A weak fluorescence for Aβ_3–16_ immunoreactivity (green) in the Con group (b) increased following LPS treatment (e) and was completely abolished by Pl administration (h). The Aβ_3–16_ fluorescence merged with the NeuN immunoreactivity, indicating the intracellular localization of Aβ (f). Scale bar: 50 μm. Aβ, β-amyloid; Con, control; i.p., intraperitoneal; LPS, lipopolysaccharide; PFC, prefrontal cortex; Pls, plasmalogens.

The presence of LPS-induced amyloidogenesis in the PFC and hippocampus following LPS injection was investigated. As shown in Figure 6, a weak fluorescence for Aβ_3–16_ immunoreactivity (green) in the PFC of the control group (Con, b) was apparently increased in the LPS group (e). The increase in Aβ_3–16_ immunoreactivity was attenuated in the LPS + Pls group (h). Neurons in the cortex were stained with NeuN (red, a, d and g), and most of the Aβ_3–16_ fluorescence merged with the NeuN immunoreactivity, indicating intracellular localization of Aβ (yellow in f).

**Figure 7:**
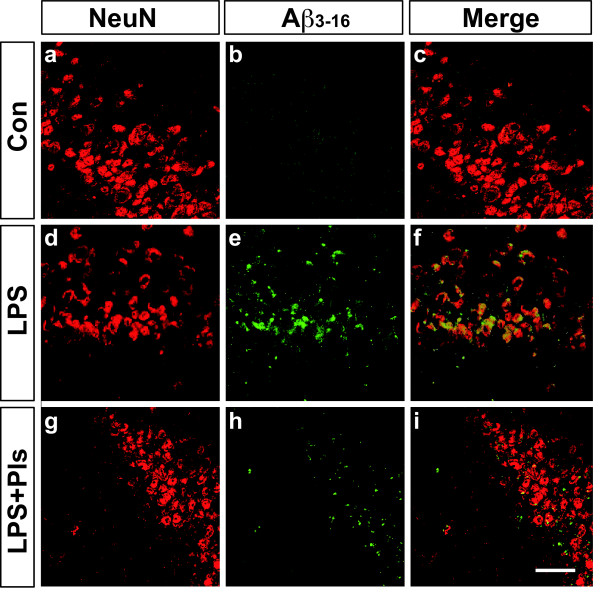
**Accumulation of Aβ proteins following LPS (i.p.) injection and suppression by Pls in the murine CA1 region of the hippocampus.** Neurons were stained with NeuN (red). A weak fluorescence for Aβ_3–16_ immunoreactivity (green) in the Con group (b) increased following LPS treatment (e) and was markedly attenuated by Pl administration (h). The Aβ_3–16_ fluorescence merged with the NeuN immunoreactivity, indicating the intracellular localization of Aβ (f and i). Scale bar: 50 μm. Aβ, β-amyloid; Con, control; i.p., intraperitoneal; LPS, lipopolysaccharide; Pls, plasmalogens.

Accumulations of Aβ were also observed in the CA1 region of the hippocampus in the LPS group (Figure 7, e). Similar to that observed in the PFC, enhanced Aβ_3–16_ immunoreactivity was markedly attenuated by Pl administration (LPS + Pls group, h). Again, the presence of intracellular accumulation of Aβ in the pyramidal neurons was detected using double staining for Aβ_3–16_ and NeuN (f and i).

### Changes in the Pl levels in the brain after LPS and Pl treatment

As shown in Table 2, the levels of PlsEtn were much higher than those of PlsCho both in the PFC and the hippocampus, although more than 60% of the phospholipids were diacylgylycereophospholipids (PEtn, PCho, PS and PI). The relative levels of PlsEtn in the PFC significantly changed following LPS injection (Table 2, upper, Kruskal-Wallis test, *χ*^2^(3) = 10.7, *P* <0.05). A multiple comparison analysis using the Steel test revealed that the amount of PlsEtn significantly decreased in the LPS group in comparison to that observed in the control group (*P* <0.05); however, the levels in the LPS + Pls group were not different from those observed in the control group (each group: n = 5). Therefore, the ratio of PlsEtn/PEtn significantly decreased following LPS treatment (*χ*^2^(3) = 11.5, *P* <0.01 and Steel test, *P* <0.05) and recovered after the i.p. administration of Pls.

**Table 2:** The changes in the phospholipid levels in the PFC and hippocampus following LPS and Pl injection

	**Con**	**LPS**	**LPS + Pls**	**Pls**
**1) PFC**				
PlsEtn	16.40 ± 0.59	13.41 ± 0.73*	16.29 ± 0.53	17.81 ± 0.32
PEtn	21.92 ± 0.63	22.66 ± 0.65	22.48 ± 0.26	22.65 ± 0.22
PlsCho	0.20 ± 0.03	0.19 ± 0.03	0.21 ± 0.03	0.28 ± 0.04
PCho	44.04 ± 1.12	47.73 ± 1.04	43.73 ± 0.97	46.74 ± 0.85
SM	3.72 ± 0.20	3.13 ± 0.31	3.47 ± 0.24	3.31 ± 0.15
PS	11.88 ± 0.27	11.08 ± 0.30	12.03 ± 0.22	10.58 ± 0.56
PI	1.86 ± 0.12	1.80 ± 0.07	1.87 ± 0.21	1.64 ± 0.31
PlsEtn/PEtn ratio	0.75 ± 0.02	0.59 ± 0.03*	0.73 ± 0.03	0.79 ± 0.01
**2) Hippocampus**				
PlsEtn	24.18 ± 0.64	20.29 ± 0.31*	22.88 ± 0.45	22.68 ± 0.39
PEtn	20.38 ± 0.43	22.07 ± 0.62	20.21 ± 0.41	20.80 ± 0.57
PlsCho	0.49 ± 0.06	0.42 ± 0.04	0.45 ± 0.05	0.41 ± 0.03
PCho	36.90 ± 1.74	39.39 ± 0.38	37.64 ± 1.37	39.90 ± 1.04
SM	5.24 ± 0.67	5.36 ± 0.59	5.71 ± 0.64	4.13 ± 0.68
PS	11.73 ± 0.86	10.94 ± 0.25	11.71 ± 0.61	10.59 ± 0.34
PI	1.59 ± 0.10	1.63 ± 0.16	1.72 ± 0.06	1.48 ± 0.16
PlsEtn/PEtn ratio	1.19 ± 0.05	0.92 ± 0.03*	1.14 ± 0.04	1.09 ± 0.05

The values are expressed as the mean ± SEM (n = 5) of the % level of the phospholipids, except for the last row. *, *P* <0.05 compared to the Con group (Steel test for multiple comparisons). Con, control; PCho, phosphatidyl choline; PEtn, phosphatidyl ethanolamine; PFS, prefrontal cortex; LPS, lipopolysaccharide; PI, phosphatidyl inositol; Pl, plasmalogen; PlsCho, choline plasmalogen; PlsEtn, ethanolamine plasmalogen; PS, phosphatidyl serine; SEM, standard error of the mean; SM, sphingomyelin.

As shown in the lower part of Table 2, the relative levels of PlsEtn in the hippocampus were greater than those in the PFC in any group (for example, 16.40 ± 0.59 versus 24.18 ± 0.64 in the Con group). Similar to that observed in the PFC, both the relative levels of PlsEtn and the PlsEtn/PEtn ratio were significantly reduced by LPS treatment (Kruskal-Wallis test, *χ*^2^(3) = 11.3 and *χ*^2^(3) = 11.4, *P* <0.01, and Steel test, *P* <0.05, respectively). The reduction of the PlsEtn levels in the hippocampus was also reversed by Pl administration. The levels of the other phospholipids did not show any significant changes following all of the treatments.

## Discussion

The present study demonstrated that systemic LPS-induced activation of glial cells, cytokine expression and accumulation of Aβ in the PFC and hippocampus were prevented by co-administration of purified Pls in adult mice. Furthermore, the injection of LPS induced decreases in the Pl levels in the PFC and hippocampus that were also suppressed by the administration of Pls.

### Mechanisms of LPS-induced accumulation of Aβ

It is well known that the activation of microglia and astrocytes plays an important role in neuroinflammation induced by systemic LPS by enhancing the secretion of cytokines, prostanoids, ROS/RNS and related substances. In the present study, i.p. injection of LPS for seven days induced morphological activation and increased the number of glial cells in the PFC and hippocampus (Figures 2,4). The amounts of IL-1β and TNF-α mRNAs, which are considered to be derived primarily from glial cells, also increased following LPS injection (Figure 5). Furthermore, LPS injection resulted in the intracellular accumulation of Aβ proteins in both regions (Figures 6 and 7). It has been reported that i.p. injection of LPS induces deficits in spatial learning in mice [24,25] that may be due to the enhancement of Aβ generation in the hippocampus [1].

It is known that β-secretase is involved in amyloidogenic processing of amyloid precursor proteins at the first step, while γ-secretase yields Aβ isoforms such as the more prevalent Aβ_40_ and aggregation-prone Aβ_42_ at the last step [26]. It has been recently shown that the activities of β- and γ-secretase are increased in the cortex and hippocampus following systemic injection of LPS [1]. It is possible that microglia play important roles in this phenomenon since proinflammatory cytokines, as well as ROS/RNS, released from activated microglia augment Aβ formation by upregulating β-secretase mRNA and enzymatic activity [5,27]. Microglia are activated further through receptors for advanced glycation end products (RAGE), which bind to Aβ and induce phagocytosis of Aβ, thereby amplifying the generation of ROS/RNS and cytokines [26].

### Changes in the Pl levels during neuroinflammation

In addition to observing the activation of glial cells and Aβ accumulation, we found that the Pl levels in the PFC and hippocampus decreased following LPS administration (Table 2). It is possible that the decreases in the amount of Pls during neuroinflammation are due to the anti-oxidant properties of Pls. It has been shown that the Pl-specific vinyl ether bond at the *sn*-1 of the glycerol backbone is targeted by a variety of oxidants, including ROS/RNS [10,11,13], and oxidative stress preferentially oxidizes PlsEtn over phosphatidyl ethanolamine (PEtn) [28,29], resulting in the disruption of vesicular fusion in the synaptosomes and the decrease in acetylcholine release [30]. This may at least partly explain why AD patients show decreases in Pl levels in the brain [14-16]. It has been suggested that abnormal membrane lipid compositions, namely decreases in the ratio of Pl to non-Pl ethanolamine glycerophospholipids, cause membrane instability in AD, which may contribute to amyloidogensis by cooperatively acting with amyloid cascade mechanisms [14]. Furthermore, since PlsEtn are major endogenous lipid constituents that facilitate membrane fusion of synaptic vesicles associated with neurotransmitter release [31,32], pathological and/or age-related alterations in the Pl levels may be attributed to neurological disorders including AD [7]. In accordance with this, it has been reported that decreases in the amount of Pls are closely correlated with the severity of dementia in humans [18,19].

### Neuroinflammation-Aβ-Pls loop

There seems to be a causes/consequences loop involving neuroinflammation that includes cytokine and ROS/RNS production, Aβ accumulation and decreases in the amount of Pls. LPS-induced activation of β-secretase [1], which is predominantly localized in cholesterol-rich lipid rafts [33,34], causes accumulation of Aβ proteins. Aβ-induced production of ROS/RNS that enhance lipid peroxidation [35,36] may decrease Pl levels, as mentioned above. In addition, increases in Aβ, cytokines and ROS/RNS reduce the expression of alkyl-dihydroxyacetone phosphate-synthase, a rate-limiting enzyme for Pl *de novo* synthesis, by inducing the dysfunction of peroxisomes, where Pls are biosynthesized, resulting in decreases in the Pl levels [37]. It has also been reported that TNF-α down regulates another key enzyme in Pl biosynthesis in peroxisomes, glycerol-3-phosphate-O-acyltransferase [38], and up regulates myeloperoxidase, which generates one of the reactive species, hypochlorous acid (HOCl), in the brain, targeting Pls to be oxidized [39]. Finally, Pls-PLA_2_, which degrades Pls to release DHA or ARA from the *sn*-2 position of the glycerol moiety, is possibly activated by ceramide produced under inflammatory conditions, and contributes to the loss of Pls in the brain [9,40].

It is well known that the generation and clearance of Aβ are affected by cholesterol metabolism, as evidenced by the identification of a variant gene of apolipoprotein E, a cholesterol transporter, as a major genetic risk factor for AD [26,41,42]. It has been shown that decreases in the amount of Pls induce a decreased rate of intracellular cholesterol transport from cell membranes to the endoplasmic reticulum, which increases the cholesterol levels in cell membranes [43]. Mankidy *et al.* further indicated that esterification of cholesterol, an obligate step that occurs prior to efflux from cells, is dependent upon the amount of polyunsaturated fatty acid (PUFA)-containing PlsEtn present in the membrane with increasing levels of the membrane-bound cholesterol-processing enzyme, sterol-O-acyltransferase-1 [44]. Increases in the cholesterol levels promote the secretion of Aβ [41,42,45], while depletion of cholesterol inhibits the generation of Aβ [46,47]. Furthermore, it has been shown that membrane Pls block cholesterol-mediated increases in β-secretase activity and directly increase the activity of α-secretase, which is known to promote non-amyloidogenic processing of amyloid precursor proteins [48]. Therefore, a vicious circle in which LPS-induced Aβ accumulation decreases the Pl levels, which leads to increased cholesterol levels, which further enhances the generation of Aβ may be involved in the pathological conditions of neuroinflammation.

### Ameliorative effects of Pls on neuroinflammation

In the present study, we showed that LPS-induced activation of glial cells (Figures 2,4), expression of IL-1β and TNF-α mRNAs (Figure 5), accumulation of Aβ proteins (Figures 6 and 7) and decreases in the PlsEtn levels (Table 2) in the PFC and hippocampus are all prevented by co-administration of Pls. Although the precise mechanisms underlying the effects of Pls in this study are not known, supplementation with Pls could improve pathological disorders. The most important question may be whether peripheral Pls can enter into the brain. So far, there are no reports indicating that Pls directly cross the blood–brain barrier (BBB). Therefore, it is not excluded that the anti-oxidative effects of Pls are exerted outside the brain in order to suppress primary inflammation induced by peripheral LPS. However, it has been shown that the Pls levels in sera are decreased in parallel with or even at earlier times than decreases in the brain Pl levels in AD patients [18,19]. Furthermore, our results showed that LPS-induced decreases in the Pls levels in the PFC and hippocampus are corrected with peripheral administration of Pls (Table 2). Therefore, it is possible that peripheral supplementation with Pls would have effects on the CNS by changing the Pl levels in the brain.

Another question is whether the effective molecules in our experiment are PUFAs, not Pls, which Pls must carry at the *sn*-2 position. Several lines of evidence show that *n*-3 PUFAs, such as eicosapentaenoic acid, DHA, and its derivative, neuroprotectin D1, have anti-inflammatory and neuroprotective effects [49-52]. Furthermore, DHA has been reported to suppress the production of Aβ proteins through multiple mechanisms, including inhibition of β-/γ-secretase activities and alteration of membrane cholesterol distribution [53-55]. Since the purified Pls used in the present study contained DHA and its precursor, α-linolenic acid, especially in PlsEtn (Table 1), it cannot be excluded that DHA derived from PlsEtn plays a significant role in the CNS effects of Pls. Indeed, it has been shown that DHA is synthesized from α-linolenic acid and incorporated into phospholipids in the liver then transported to the brain through the peripheral circulation [56]. On the other hand, it has also been shown that lyso-type phospholipids, which contain DHA at the *sn*-2, show preferential transfer over DHA in *in vitro* models of the BBB [57]. Furthermore, it has been suggested that specific transport mechanisms to import Pls and their synthetic precursors exist in brain capillary epithelial cells [58,59]. These findings suggest that Pls containing DHA exert more effective actions in the CNS than DHA alone.

The present study suggests that co-administration of Pls suppresses systemic LPS-induced neuroinflammation in the brain. Although further studies on the mechanisms underlying these CNS effects, including the metabolism of the administered Pls and the pathways used to enter the brain, are needed, the present results indicate that Pls may possibly be used in new preventive and therapeutic strategies for treating AD.

## Abbreviations

Aβ, β-amyloid proteins; AD, Alzheimer’s disease; ANOVA, analysis of variance; ARA, arachidonic acid; BBB, blood–brain barrier; bp, base pair; BW, body weight; CNS, central nervous system; DG, dentate gyrus; DHA, docosahexaenoic acid; GFAP, glial fibrillary acidic proteins; HOCl, hypochlorous acid; HPLC, high performance liquid chromatography; Iba-1, ionized calcium binding adaptor molecule-1; IFN-γ, interferon-γ; IgG, immunoglobulin G; IL-1β, interleukin-1β; i.p., intraperitoneal; LPS, lipopolysaccharide; NSAIDs, non-steroidal anti-inflammatory drugs; PBS, phosphate buffered saline; PCho, phosphatidyl choline; PEtn, phosphatidyl ethanolamine; PFC, prefrontal cortex; PI, phosphatidyl inositol; Pls, plasmalogens; PlsCho, choline plasmalogens; PlsEtn, ethanolamine plasmalogens; Pls-PLA2, plasmalogens-selective phospholipase A2; poly I:C, polyriboinosinic:polyribocytidylic acid; PPAR-γ, peroxisome proliferator-activated receptor-γ; PS, phosphatidyl serine; PUFA, polyunsaturated fatty acid; RAGE, receptors for advanced glycation end products; ROS/RNS, reactive oxygen/nitrogen species; SM, sphingomyelin; TLR, toll-like receptor; TNF-α, tumor necrosis factor-α.

## Competing interests

The authors declare that they have no competing interests.

## Authors’ contributions

MI prepared the manuscript and performed the behavioral tests, immunohistochemistry and real-time PCR. TK designed the studies, performed the statistical analysis and wrote the manuscript. SM measured the levels of the brain Pls and analyzed the fatty acid composition of the Pls. MN assisted with manuscript preparation and discussed the data. KM and MS together prepared the purified Pls. TF designed the studies and reviewed and discussed the data. All authors read and approved the final manuscript.
